# Improving the Diagnostic Accuracy of the PD-L1 Test with Image Analysis and Multiplex Hybridization

**DOI:** 10.3390/cancers12051114

**Published:** 2020-04-29

**Authors:** Matthew P. Humphries, Victoria Bingham, Fatima Abdullahi Sidi, Stephanie G. Craig, Stephen McQuaid, Jacqueline James, Manuel Salto-Tellez

**Affiliations:** 1 Precision Medicine Centre of Excellence, The Patrick G Johnston Centre for Cancer Research, Queen’s University, Belfast BT9 7BL, UK; m.humphries@qub.ac.uk (M.P.H.); v.bingham@qub.ac.uk (V.B.); F.AbdullahiSidi@qub.ac.uk (F.A.S.); Stephanie.Craig@qub.ac.uk (S.G.C.); s.mcquaid@qub.ac.uk (S.M.); j.james@qub.ac.uk (J.J.); 2 Cellular Pathology, Belfast Health and Social Care Trust, Belfast City Hospital, Lisburn Road, Belfast BT9 7BL, UK; 3 Northern Ireland Biobank, The Patrick G Johnston Centre for Cancer Research, Queen’s University, Belfast BT9 7BL, UK

**Keywords:** PD-L1, clinical workflow, multiplexing, image analysis

## Abstract

Targeting of the programmed cell death protein (PD-1)/programmed death-ligand 1 (PD-L1) axis with checkpoint inhibitors has changed clinical practice in non-small cell lung cancer (NSCLC). However, clinical assessment remains complex and ambiguous. We aim to assess whether digital image analysis (DIA) and multiplex immunofluorescence can improve the accuracy of PD-L1 diagnostic testing. A clinical cohort of routine NSCLC patients reflex tested for PD-L1 (SP263) immunohistochemistry (IHC), was assessed using DIA. Samples of varying assessment difficulty were assessed by multiplex immunofluorescence. Sensitivity, specificity, and concordance was evaluated between manual diagnostic evaluation and DIA for chromogenic and multiplex IHC. PD-L1 expression by DIA showed significant concordance (R² = 0.8248) to manual assessment. Sensitivity and specificity was 86.8% and 91.4%, respectively. Evaluation of DIA scores revealed 96.8% concordance to manual assessment. Multiplexing enabled PD-L1+/CD68+ macrophages to be readily identified within PD-L1+/cytokeratin+ or PD-L1-/cytokeratin+ tumor nests. Assessment of multiplex vs. chromogenic IHC had a sensitivity and specificity of 97.8% and 91.8%, respectively. Deployment of DIA for PD-L1 diagnostic assessment is an accurate process of case triage. Multiplex immunofluorescence provided higher confidence in PD-L1 assessment and could be offered for challenging cases by centers with appropriate expertise and specialist equipment.

## 1. Introduction

Durable tumor regression and prolonged stabilization of disease in patients treated with immune checkpoint blockade therapy has changed the paradigm with cancer immunotherapy. The expression of programmed death-ligand 1 (PD-L1) has been associated with profound responses to anti– programmed cell death protein (PD-1) therapy and led to several U.S. Food and Drug Administration approved PD-L1 diagnostic assays for melanoma, non–small cell lung cancer (NSCLC), and gastric, bladder, and cervical cancers [1,2,3,4]. However, the accelerated adoption of these diagnostic tests has highlighted several difficulties in the pathological assessment of PD-L1. We recently reported on the routine challenges faced clinically in assessment of PD-L1 [5]. This is in addition to the myriad of companion diagnostic assays available for PD-L1 and the variation in assessment criteria across tumor types [6,7,8]. The imperfect nature of PD-L1 testing highlights concerns over inter- and intra-laboratory variations when assessing PD-L1 expression, raising questions on the reproducibility of the tests among pathologists [9]. 

Approval of companion diagnostic tests assumes a robustness, precision, and reproducibility for deployment in accredited laboratories across the world. However, the expression of PD-L1 in tumor cells does not always identify NSCLC patients that would benefit from an immune checkpoint blockade, indeed, high PD-L1 tumor cell expression does not consistently predict a favorable clinical response [10,11,12].

The application of digital image analysis (DIA) to digital PD-L1 immunohistochemistry (IHC) slides has the potential to improve the accuracy and reproducibility of the diagnostic test. However, DIA is hindered, as is the pathologist, by the sharp clinical thresholds, intrinsic macrophage staining, presence of positive PD-L1 inflammatory cells around nests of malignant epithelium (the so called “hugging effect”), and the occasional poor delineation of specific tumor cells, particularly in cytology samples [5]. 

Recently, efforts to assess multiple proteins within the tumor microenvironment in relation to PD-L1 have yielded improved predictive power in clinical performance to immunotherapy, comprehensively summarized by S. Lu et al. [13]. The superior diagnostic accuracy is attributed to the ability to accurately assess the co-expression of multiple biomarkers simultaneously, in the specific cell types expressing PD-L1, while retaining their spatial relationships.

While these studies have focused on the prognostic and predictive value of multiple biomarker assessment, to our knowledge, no studies to date have successfully demonstrated the utility of a PD-L1 immunofluorescent multiplex assay, utilizing clinically relevant clones, on routine diagnostic cases, to enhance and improve the clinical accuracy of PD-L1 assessment. 

Here, we present a comprehensive assessment of PD-L1 IHC using DIA on NSCLC reflex tested cases. We demonstrate the concordance with manual pathological assessment, evaluate the potential for DIA utilization in routine clinical diagnostics, the reasons for clinical discordance, and recommendations for PD-L1 case triage. Importantly, we describe the practicality and effectiveness of a clinically deployable PD-L1/cytokeratin(CK)/CD68/CD8/DAPI multiplex as a viable lab-developed test for the evaluation of PD-L1 reflex tested cases in an accredited laboratory. 

## 2. Results

### 2.1. PD-L1 Testing in Routine Practice

**Figure 1:**
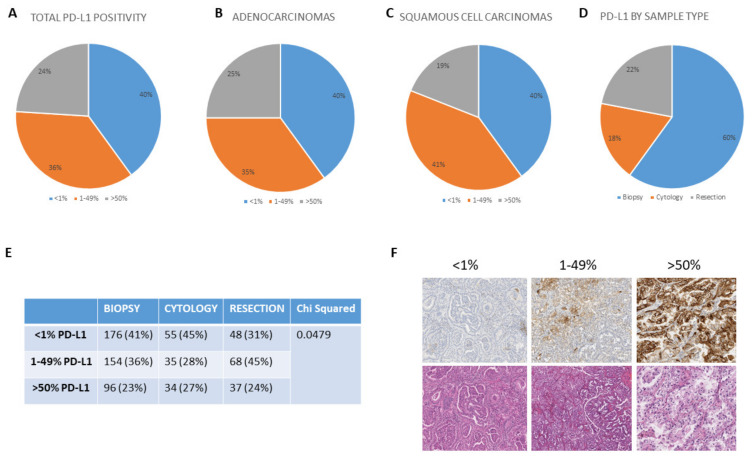
Comparable categorical distribution of programmed death ligand 1 (PD-L1) expression in (**A**) 703 clinical cases, (**B**) Adenocarcinomas, (**C**) Squamous cell carcinomas and (**D**) Sample types. (**E**) Shows the categorization of the PD-L1 expression according to sample type. The *p* value is determined by the chi-square test. (**F**) Left-to-right display representative images of <1%, 1–49% (×10 magnification) and >50% (×20 magnification) PD-L1 expression, with the corresponding tumor hematoxylin and eosin (H&E) below.

There were 703 cases submitted for PD-L1 analysis and had clinical reports issued. Of these, 40% were PD-L1 negative (<1% positive), with 36% reported as 1–49% and 24% reported as >50% PD-L1 positive (Figure 1A). Adenocarcinomas and squamous cell carcinomas showed little difference in PD-L1 categorization (Figure 1B,C). The PD-L1 positivity by sample types is shown in Figure 1D and includes 60% biopsies, 18% cytologies, and 22% surgical resections. Figure 1E shows the PD-L1 expression according to sample type, with *p* value determined by the chi-square test. In line with our previous observations [5], we found a significantly different (*p* = 0.0479) distribution of PD-L1 IHC cases in the 1–49% category in resection specimens than either <1% or >50%, indicating that in resection cases, patients are disproportionally likely to be categorized as 1–49% PD-L1 positive. Representative PD-L1 categories are shown in Figure 1F as well as the corresponding hematoxylin and eosin (H&E) images.

### 2.2. Concordance of Image Analysis and Manual PD-L1 IHC Assessment 

**Figure 2:**
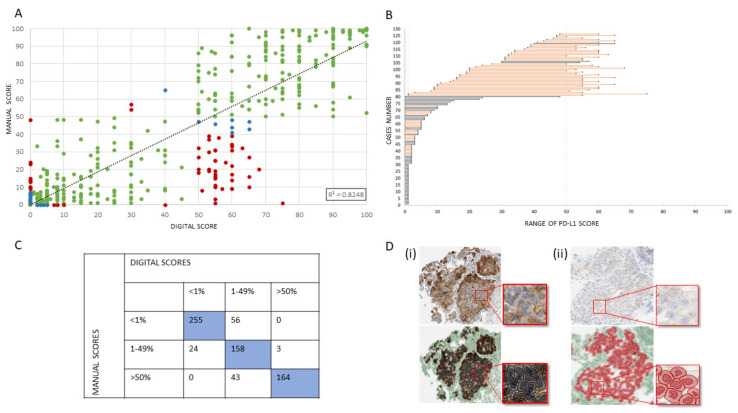
Concordance of manual PD-L1 assessment with digital pathology. (**A**) Correlation of scores by the two methodologies. Categorical agreement is represented by green data points; acceptable discordance by blue data points; and unacceptably discordance cases by red data points. (**B**) The range of discordance across the clinical thresholds for each of the 126 discordant cases. Data points specify a PD-L1 score. Black connecting lines connect a lower digital scores to a higher manual score, while an orange line connects a lower manual score to a higher digital score. (**C**) Categorical concordance and discordance in terms of total numbers. (**D**) (i) Concordant comparison between manual and digital assessment in a case which was high for PD-L1 expression. Figure 2D (ii) A non-concordant comparison from a low PD-L1 expressing case. In these examples, the image analysis mask describes PD-L1+ tumor cells in black and PD-L1- tumor cells in red, with stromal cells shown in green. Images are ×4 magnification with an exploded view of a higher magnification area at ×40 shown.

Manual PD-L1 assessment (the current gold standard) and QuPath DIA were highly correlated, R² = 0.8248 as shown in Figure 2A, with a sensitivity and specificity of 86.8% and 91.4%, respectively. In 82% of clinical cases (577/703), both assessments were fully concordant, while 18% (126/703) of clinical cases were discordant (Figure 2B). In 56 cases, manual assessment was <1%, while the digital assessment was 1–49%. For 27 cases, manual assessment was 1–49%, while the digital score was <1% (*n* = 24) or >50% (*n* = 3). Forty-three cases scored as >50% by manual assessment were scored as 1–49% by digital analysis (Figure 2C). The concordance between manual and digital assessment by sample type and histology is shown in Figure S1. Figure 2D (i) shows a concordant comparison between manual and digital assessment in a case which had >50% PD-L1 expression. Figure 2D (ii) shows a non-concordant comparison from a 1–49% PD-L1 expressing case. Within the specific scoring ranges of 10–49% and >70%, DIA had a concordance of 96.8%.

### 2.3. Challenges of Image Analysis on Routine PD-L1 IHC

All discordant cases (*n* = 126, Figure 2B) were visually reviewed. Of those, 73 cases were found to be acceptably discordant due to the objective ground truth being difficult to establish (Figure 2A; blue data points), and having an average standard deviation of 2.6%. Fifty-three of those cases were considered truly discordant (Figure 2A; red data points). The main reasons for discordance between manual and digital assessment were difficult classification of tumor cells by DIA (particularly in cytology samples); overabundance of macrophages; spurious staining inclusion; and lower threshold sensitivity (particularly in squamous cell carcinoma cases). The number of cases in each discordant group are detailed in Table 1. Cases that were acceptably discordant were focused around the clinical thresholds of 1% and 50% (typically <5% or between 40% and 60%). The range of discordance across the clinical thresholds for each of the 126 cases is detailed in Figure 2B. 

### 2.4. Comparative Analysis and Utility of PD-L1 Multiplexing 

**Figure 3:**
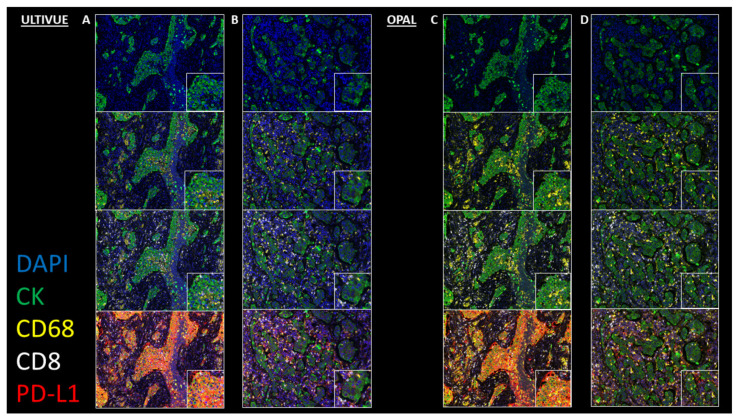
Representative images of comparative multiplex methodologies. High PD-L1 expressing case: (**A**) ULTIVUE (**C**) OPAL. Low PD-L1 expressing case: (**B**) ULTIVUE (**D**) OPAL. In each column of images, progressive channels are included. From top to bottom, images contain DAPI+/CK+ initially (blue/green), followed by addition of CD68 (yellow), CD8 (white), and PD-L1 (red). Each image includes a high powered magnification field of view (×20 magnification). In all composite images (×8 magnification), PD-L1+/CD68+ and PD-L1+/CD8+ cells are clearly distinguishable in the tumor bed.

**Figure 4:**
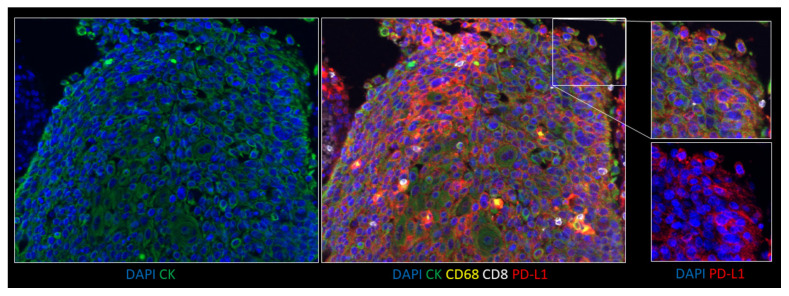
Representative image of fine membrane staining in a squamous cell carcinoma by ULTIVUE multiplex at ×10 magnification. An exploded view of a higher magnification area is shown on the right (×20 magnification) with the composite above and DAPI/PD-L1-only channel below.

In biopsy, cytology, and resections for both adenocarcinoma and squamous cell carcinoma samples, immunofluorescence staining by ULTIVUE and OPAL multiplex methods showed specific and sensitive PD-L1 expression within the range of expected cell types (tumor epithelium, macrophages, and immune cells). For tumor epithelium, PD-L1 expression ranged from absent through to very strongly positive in individual samples. CK, CD68, and CD8 expression levels were also evaluated as similar by both multiplex methods. Each method performed equally well in multichannel mode with clear resolution of PD-L1+/CK+, PD-L1+/CD68+, and PD-L1+/CD8+ cells (Figure 3). Importantly, for both methods (ULTIVUE, Figure 3A,B and OPAL, Figure 3C,D), PD-L1+/CD68+ macrophages could be readily identified within nests of strongly positive PD-L1+/CK+ (Figure 3 column A,C) or negative PD-L1-/CK+ tumor cells (Figure 3 column B,D). The presence of autofluorescence was marginally apparent within each sample by both methods, however, the strength of the individual biomarker signals was such that autofluorescence was easily discounted from visual assessments. This was particularly relevant in the assessment of fine membrane staining on some tumor cell populations, especially in squamous cell carcinomas (Figure 4). The morphological detail of the sections was not compromised by either multiplex method.

In the 156 samples assessed in the comparison of ULTIVUE and OPAL multiplex assays, ULTIVUE was in 99% concordance with IHC, whilst with OPAL concordance was 93%. However, we would caution over-interpretation of this comparison due to the subjective nature of PD-L1 assessments. Most of the OPAL discordant cases were in the 0–2% category and pathologist concordance as much as technical discordance should be considered. The ULTIVUE UltiMapper I/O PD-L1 multiplex method was therefore taken forward as the multiplex of choice for comparison with the gold standard diaminobenzidine (DAB) PD-L1 for reasons of concordance and the following operational considerations: (1) lower technical complexity of the test in the laboratory, (2) fewer component reagents for the user to prepare, (3) fewer retrieval steps required, (4) speed of the automated staining run (5 h; opposed to 12 h for OPAL), and (5) no requirement for complex in-house pre-validation.

Blinded to the clinical data and the DAB PD-L1 score, a comparative manual assessment of DAB PD-L1 against the ULTIVUE UltiMapper I/O PD-L1 multiplex was conducted. In 330 biopsy, cytology, and resections of both adenocarcinoma and squamous cell carcinoma samples, the sensitivity of the multiplex was 97.8% and specificity of the assay was 91.8%, as calculated by data shown in Table 2. Discordances between the two methodologies were, in some instances, due to very weak staining visualized with DAB, where multiplex was able to provided more discernible positivity. Equally, an example where a DAB PD-L1 score was 20% but assessed as 5% by multiplex was due to an enhanced delineation of CK+/PD-L1 cells in the multiplex, increasing the denominator and lowering the overall score. In a cytology specimen, DAB assessment resulted in a score of <1% (negative), however, multiplex concluded that <100 CK+ tumor cells were present in the sample (and therefore clinically inadequate). The main cell type present was CD68+ macrophages, which were easily misconstrued as negative tumor cells rather than an inadequate sample using brightfield assessment.

**Figure 5:**
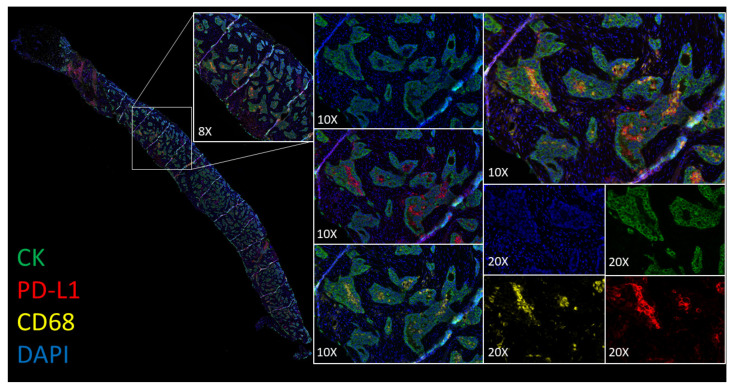
Utility of a PD-L1 multiplex on the assessment of a diagnostic case. Whole slide image of a strip biopsy with ×8 and ×10 magnifications of a region of interest. The ×10 magnifications show (top to bottom) DAPI/CK, DAPI/PD-L1, and DAPI/CD68. A ×20 magnification, inclusive of each individual channel of diagnostic interest, is shown on the right. Within distinct tumor beds, the majority of the positive cells are of a PD-L1+/CD68+ phenotype and are not PD-L1+/CK+.

The beneficial utility of a PD-L1 multiplex on the assessment of a diagnostic case is shown in Figure 5 and Video 1, demonstrating the capacity to confidently assess the PD-L1 positivity in PD-L1+/CK+ cells or PD-L1-/CK+ cells while having the ability to discount PD-L1+/CD68+ macrophages. 

**Figure 6:**
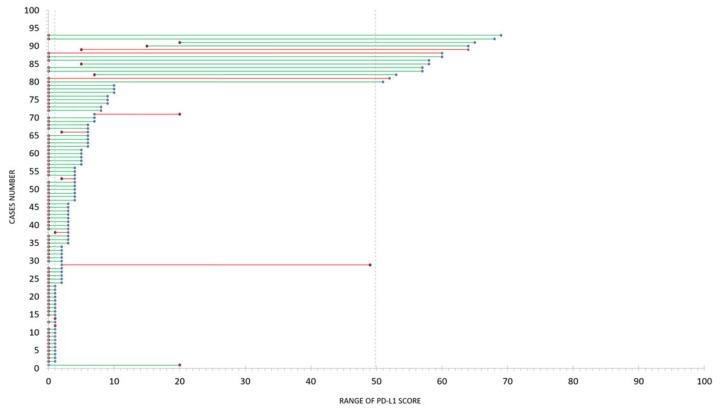
The accuracy of multiplex in determining PD-L1 scores in discordant samples. Ninety-three discordant samples close to the clinical threshold assessed by manual pathology review and by QuPath image analysis are shown on the Y-axis. Blue data points specify a PD-L1 score determined by QuPath image analysis. Red data points specify a PD-L1 score determined by multiplex. Green connecting lines connect the discordant image analysis score to a multiplex score which agreed with the manual pathology review, while a red line connects a discordant image analysis score to a multiplex score that remained discrepant to the manual pathology review.

Samples that were discordant between manual and digital assessment, within a window of uncertainty (PD-L1 scores of <10% and 50–70%), were assessed by ULTIVUE I/O PD-L1 multiplex, Figure 6. In 83/93 instances, where image analysis was discrepant with the manual score, multiplex was able to accurately ascribe the same PD-L1 clinical category as was determined manually (Figure 6). In 10/93 samples the multiplex score did not agree with the manual assessment and was either in agreement with the DIA (7/10) or determined that the score should fall within a different clinical category than either the manual or DIA findings (3/10), Figure 6. Discordant samples at <10% were successfully rescued by multiplex to an agreement with the manual pathologist assessment 91% of the time.

## 3. Discussion

Here, we describe our experience of and the challenges and future opportunities of DIA on PD-L1 IHC testing. Notably, we report on the validation of a clinically deployable PD-L1 multiplex as a lab-developed referral test. 

Building upon our previous observations [5], where PD-L1 expression patterns seen in resections do not mirror exactly that seen in the cytology and biopsy samples, we identified a significant increased PD-L1 expression in the 1–49% category of resection specimens, likely borne out by the increased *n* number in the present study. This is explainable by the observation that increased tissue area for assessment leads to an increased reporting of 1–49% cases from resection specimens. This is possibly due to the difficulty of microscopically assessing large areas of tissue that extend beyond a single field of view and, therefore, to precisely calculate the total percentage of tumor and the total positive tumor. This observation indicates that patients assessed on resection specimens could be more likely to receive 2nd line treatment rather than be categorized as <1%. Additionally, the even balance of PD-L1 positivity across the samples types demonstrated a robustness of the 50% threshold to dictate 1st line treatment. 

The complexity and ambiguity of the assessment of the PD-L1 diagnostic test is well reported [5,13,14]. The large variation in antibody clones, staining platforms, and assessment criteria plague pathology departments globally. Leading on from others, as well as our own comprehensive assessment of PD-L1 IHC [5], we demonstrate herein the potential role of digital pathology in the automated scoring of PD-L1. 

Our experience in 703 cases indicates that there is a high degree of concordance between manual pathological evaluation and digital analysis. Several considerations are highlighted in the quality control steps required for diagnostic deployment of DIA on PD-L1 IHC: (1) Confirming tumor classification accuracy, (2) Excluding abundant macrophage presence, (3) Avoiding slides with large areas of spurious staining, and (4) Confirming lower threshold sensitivity levels. As a result, a policy of mandatory pathologist evaluation should be implemented when a digital score approaches 10% of a clinical threshold. Our recommendation for mandatory pathologist review would fall to cases digitally scored at <75%, as in our experience, the accuracy of DIA was 100% concordant above 75%. That being said, DIA was highly accurate between 10–49% and >70%. Cases close to a diagnostic threshold consistently required more detailed review, and as such a sliding scale of pathologist confidence in the DIA result may represent the most beneficial use of DIA in case triage. This could be represented by an authorized digital score with a degree of confidence in the assessment, on a case by case basis. In such a triage situation, for the assessment of positive cases only (10–49% and >70%), the absence of negative cases makes a calculation of specificity logically impossible. These data suggest that an optimal analytical window exists whereby digital assessment is achievable and highly reliable.

Discordant cytology cases were more likely to be truly discordant than acceptably discordant, and the disagreement was predominantly where a higher manual assessment was reported. Whether this represents an ability of DIA to accurately calculate the tumor cell denominator better than a pathologist, or is alternatively a failing of DIA to accurately classify cytology specimens due to the lack of tissue architecture usually required when attempting to build robust classifiers is debatable.

It is important to highlight the need for suitable slides for accurate DIA. Algorithms associated with DIA have a low tolerance for poor section quality, which manual microscopic assessment permits, meaning, in the present study, fewer samples met our minimum required criteria for assessment for DIA compared to manual assessment. Moreover, the minimum and maximum time taken to assess a whole slide image may vary greatly due to many characteristics of the sample, as described. In particular, time taken to analyze a slide can be largely dictated by a vast array of in silico factors, e.g., the gigapixel size of the images or local computational specifications. Biopsy samples for example can be represented by a few cells (in the low hundreds), which necessitate less processing time, whereas large resections can contain millions of cells, requiring more time to process during DIA. 

When confidence in ascribing a PD-L1 score to a DAB IHC slide is challenging, the application of multiplex could be beneficial in specific cases where cell type specific PD-L1 assessment is extremely difficult. Based on our experience, a diagnostic decision tree is useful in proposing the most beneficial application of DIA and multiplex to appropriately triage cases (Figure S2). 

While we did observe discordance between multiplex and single-plex IHC, multiplexing provides a higher level of confidence in the identification of specific cell types present in samples and, therefore, an increased assurance in the overall PD-L1 score reported. The authors of a recent meta-analysis on PD-L1 multiplexing concluded, as do we, that multiplex appeared closer to the truth when determining PD-L1 positivity [13]. Furthermore, they postulated that multiplex was able to accurately identify the nature of the cellular co-expression of PD-L1 and was consequently more predictive of response to immunotherapy. Larger multiplex studies in cohorts of immunotherapy treated patients may yet yield greater insight into the varying response rate seen in clinical trials across several cancer types. 

It is important to recognize that a sensitivity and specificity analysis used to assess the suitability of a test (multiplex) to a gold standard (PD-L1 DAB IHC) can only be as reliable as the reference test is capable of determining sample status without error [15]. A recent publication assessed the sensitivity and specificity of image analysis to the pathologist gold standard in 100 cases and their findings showed, as have other studies, that automated scoring was no worse than the concordance between pathologists [14,16]. As PD-L1 IHC is an imperfect test, where no other reference test or standards are available that fully confirm the pathologist’s subjective score [9], a full comprehensive validation is required to verify multiplexing accuracy. This should include critical, clinical performance parameters relevant to the specific technology to provide the highest chance of detecting sources of variation and interference [15]. In our opinion, this lack of certainty in PD-L1 assessment calls for the development of reference materials for multicenter validation, over and above the quality of staining assessments of accreditation bodies such as the College of American Pathologists and other national accreditation programs. 

In future, as for DAB PD-L1 IHC, DIA has the potential to aid in the evaluation of PD-L1 multiplex. Such feasibility warrants further important investigation in large cohorts of cases where multiplex has been applied, especially in cohorts of immunotherapy treated patients. 

## 4. Materials and Methods

### 4.1. Clinical Samples

Eight hundred two cases were submitted for diagnostic PD-L1 assessment as a reflex test over a 20 month period from four regional Northern Ireland hospitals (North, North-Western, South-Western, and Belfast Trusts) to the Regional Diagnostic Molecular Pathology Laboratory. Of the 802 cases, 99 were unsuitable for PD-L1 testing due to the sample containing <100 tumor cells or were, after central review, of an inappropriate cancer type. The remaining 703 NSCLC cases were reflex tested and had reports issued. Sample types included formalin fixed paraffin embedded (FFPE) blocks, bronchoscopic and core biopsies (*n* = 426), cytologies (*n* = 124), and surgical resections (*n* = 153). We have previously reported demographics for a large proportion of our lung cohort (564 cases) [5]. Additional whole slide images from our routine service were collected. Our cohort, from the same source of tissue samples, followed the exact same trends in terms of PD-L1 distribution and the key descriptors were the same, this is evidenced by the equivalent spread of the data in Figure 1. All cases were manually assessed and a consensus score reported by teams of two individuals who received training and are certified competent for clinical scoring of PD-L1 in NSCLC. To assess specificity and sensitivity, an intra-run reproducibility section from a four core tissue microarray was used in each test run, representing PD-L1 expression levels of <1%, 1–49% and >50%, as well as a positive control (tonsil). Stained tumor slides and blocks were retrieved and provided via the Northern Ireland Biobank, which has ethical approval to use de-identified tissue samples from the Belfast Health and Social Care Tissue Pathology archive (REC:11/NI/0013). 

### 4.2. Routine Diagnostic Staining

Sequential 3µm sections were obtained from FFPE tumor blocks and used for routine diagnostic IHC on biopsy, cytology, and resection samples, with a section for H&E also obtained. IHC was performed using an automated staining system (Ventana BenchMark, Roche Diagnostics, Basel, Switzerland) with a PD-L1 SP263 clone with a locked-in protocol as recommended by the company (Ventana, CC1 pre-treatment for 64 mins, Ventana Optiview detection protocol), a DAB reaction was used to detect antibody labelling with hematoxylin counterstaining. 

### 4.3. PD-L1 IHC Image Analysis 

DIA of all DAB PD-L1 SP263 IHC stained cases was performed using the open source DIA program QuPath v0.1.2, developed at Queen’s University Belfast [5,17,18,19,20]. All IHC slides were scanned at 40× on an Aperio AT2 digital scanner (Leica Biosystems, Vista, CA, USA). A robust workflow and rigorous quality control steps were taken to remove unsuitable areas for analysis (e.g., necrosis, tissue folds, normal structures, and non-specific staining), this was confirmed by a second reviewer with frequent consultation, as described [5,17,18,19,20]. Briefly, digital annotations were made, within which cell detection was conducted using default parameters within QuPath. Annotations were made on the whole slide image by an experienced image analyst, encompassing the tissue to be analyzed within a single region of interest under the supervision of an experienced clinician prior to analysis. Classification of cell types was applied, using the random forest method, to distinguish tumor and stroma compartments under the consultation of pathologists experienced in PD-L1 clinical assessment. A positive cell was defined as a tumor epithelial cell that showed a pattern of membrane staining, complete or partial, of any intensity, classified by specific features, within the class (tumor), above a DAB threshold of 0.015, determined to be the lower limit of positive detection by clinical expertise. Sensitivity and specificity calculations were based on the following equations: (True Positive/(True Positive + False Negative)×100 and True Negative/(True Negative + False Positive) × 100, respectively. 

### 4.4. Multiplex Staining

Three hundred thirty additional lung tumor samples were selected for a range of PD-L1 tumor expression patterns and for varying degrees of PD-L1 expression on macrophages and/or other cell types, enriching the cases of potential diagnostic difficultly. Sections were stained with validated methods for routine diagnostic DAB PD-L1, as described. In 156 of the 330 samples, on sequential sections, a comparison of two validated multiplex methodologies was conducted using Opal 7-Color Automation IHC Kit (PD-L1/CK/CD68/CD8/DAPI) (Akoya Biosciences, Marlborough, MA, USA) and ULTIVUE UltiMapper I/O PD-L1 multiplex immunostaining kit (PD-L1/CK/CD68/CD8/DAPI) (ULTIVUE; Cambridge, MA, USA), conducted on a Leica Bond Rx fully automated immunostainer. Optimized retrieval methods and staining steps for Opal and ULTIVUE were used according to the manufacturer’s instructions and are detailed in Table S1. All multiplex slides were scanned on a Vectra Polaris (Akoya Biosciences) at ×20.

Post validation and qualitative evaluation in multichannel format of both multiplex methodologies were conducted. Criteria used in the evaluation of each multiplex method were (1) concordance with DAB PD-L1, (2) resolution and specificity of PD-L1, CK, CD68, and CD8 reactivity in single-channel and multichannel mode, (3) presence of autofluoresence in individual channels, and (4) morphological integrity of the sections. Following acceptance of one multiplex methodology, further staining and assessment in all 330 lung NSCLC samples was conducted.

## 5. Conclusions

The application of digital pathological DIA in clear-cut PD-L1 cases could enable the streamlining of the pathology workflow, allowing more time-consuming cases to be the main focus of the pathologist. While we do not advocate that PD-L1 DAB IHC should be replaced by multiplexing as a new gold standard in clinical practice, we propose, that in very challenging cases, a multiplex could be offered as a specialist test in centralized centers of excellence that have access to the expertise and specialist equipment required to fully deploy and assess this methodology. 

## Figures and Tables

**Table 1:** Reasons for discordance.

Reasons for Discordance	Number of Cases
Classifier	22
Macrophages	8
Spurious Staining	41
Threshold sensitivity	55

**Table 2:** Sensitivity and specificity data.

		PD-L1 DAB IHC		
		Positive	Negative	Total
**PD-L1 Multiplex**	**Positive**	141	15	156
	**Negative**	4	170	174
	**Total**	145	185	330

## Supplementary Materials

The following are available online at https://www.mdpi.com/2072-6694/12/5/1114/s1, Figure S1: Concordance between Manuel and digital assessment. Figure S2: A diagnostic decision tree proposing the most beneficial application of image analysis and multiplex. Table S1: Antibody and Retrieval Information. Video S1: Utility of a PD-L1 multiplex on the assessment of a diagnostic case.
