# Supplementary material for: Improving the Diagnostic Accuracy of the PD-L1 Test with Image Analysis and Multiplex Hybridization
Source: Cancers (Basel). 2020 Apr 29;12(5):1114. doi: 10.3390/cancers12051114 (PMC7281311; doi:10.3390/cancers12051114)
Supplement: Supplementary file 1 [file cancers-12-01114-s001.zip › cancers-763243-suppl xml.pdf]

cancers

Supplementary Materials: Improving the Diagnostic Accuracy of the PD-L1 Test with Image Analysis and Multiplex Hybridization

Table S1. Antibody and Retrieval Information.

**DAB Immunohistochemistry—Ventana Benchmark XT**							
Biomarker	Clone	Source	Antigen Retrieval	Dilution	Incubation	Detection chemistry	Control tissue
PD-L1	SP263	Ventana	CC1 for 64 minutes	Neat	20 minutes	OptiView DAB	Tonsil

**Opal Multiplex Immunofluorescence – Leica Bond RX**									
Step	Biomarker	Clone	Source	Antigen Retrieval	Dilution	Incubation	Detection chemistry	Incubation	Control tissue
1	CD8	C8/144B	Dako	ER2 20 minutes @ 95°C	1:400	30 minutes	Opal 690 @ 1:150	30 minutes	Tonsil
2	PD-L1	SP263	Ventana	ER2 20 minutes @ 95°C	1:2	30 minutes	Opal 520 @ 1:150	30 minutes	Tonsil
3	CD68	514H12	Leica	ER2 20 minutes @ 95°C	1:200	30 minutes	Opal 570 @ 1:150	30 minutes	Tonsil
4	CK	AE1/AE3	Dako	ER2 20 minutes @ 95°C	1:100	30 minutes	Opal 620 @ 1:150	30 minutes	Tonsil
5	DAPI	n/a	Perkin Elmer	ER2 20minutes @ 95°C	1:500	10 minutes	n/a	n/a	Tonsil

**ULTIVUE Multiplex Immunofluorescence – Leica Bond RX**								
Step	Reagent	Clone	Source	Antigen Retrieval	Dilution	Incubation	Detection chemistry	Control tissue
1	Antibody Diluent	n/a	Ultivue	n/a	neat	15 minutes	n/a	Tonsil
2	PD-L1, CD8, CD68 & CK	7310, C8/144B, KP-1, & AE1/AE3	Ultivue	ER2 20 minutes @ 95°C	1:100	60 minutes	n/a	Tonsil
3	Pre-Amplification Mix	n/a	Ultivue	n/a	neat	25 minutes	n/a	Tonsil
4	Amplification Solution	n/a	Ultivue	n/a	1:10	90 minutes	n/a	Tonsil
5	Nuclear Counterstain	n/a	Ultivue	n/a	1:100	15 minutes	n/a	Tonsil
6	Fluorescent Probes	n/a	Ultivue	n/a	1:20	25 minutes	Cy5, FITC, Cy3, Cy7.	Tonsil

All incubations at room temperature unless otherwise stated.

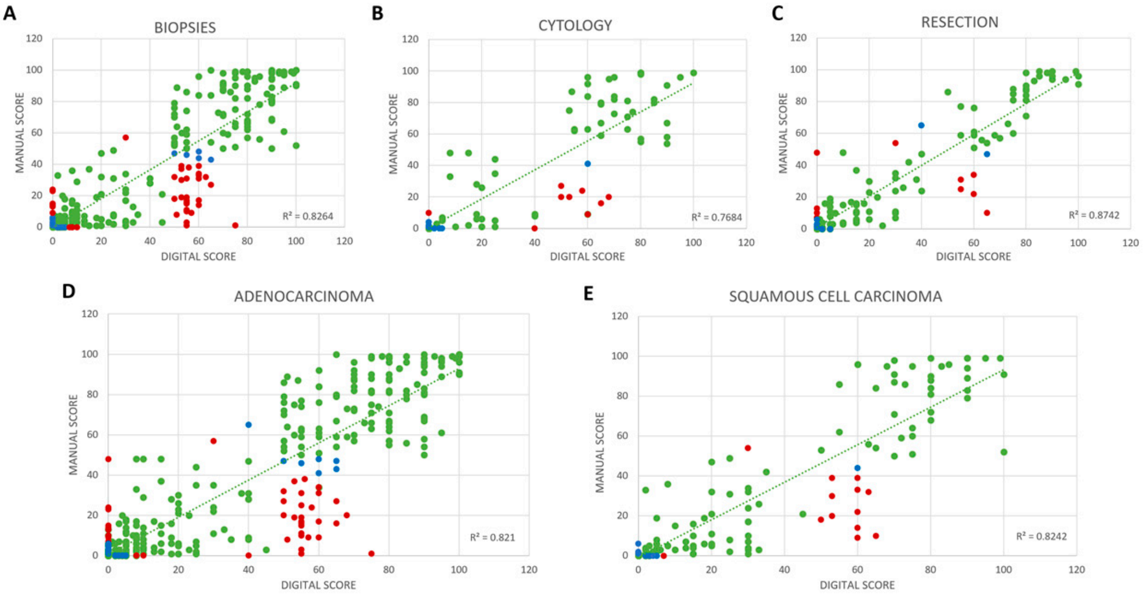

Figure S1. Concordance between Manuel and digital assessment. Concordance between manual and digital assessment by (A) Biopsy (B) Cytology (C) Resections and histology (D) and (E). In all the same types and both adenocarcinoma and squamous cell carcinoma samples there is a high level of concordance.

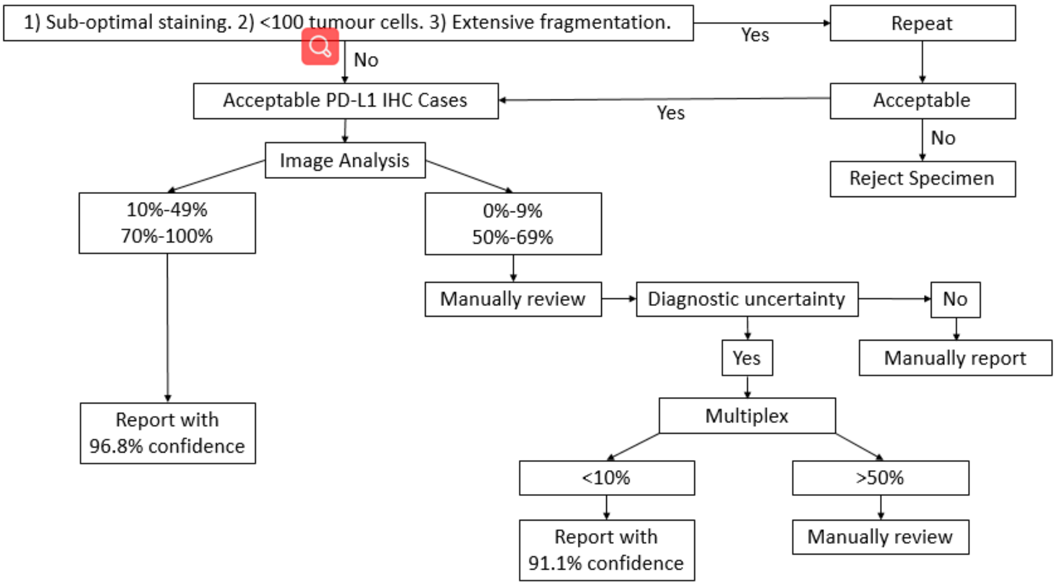

Figure S2. A diagnostic decision tree proposing the most beneficial application of image analysis and multiplex to appropriately triage PD-L1 diagnostic cases.
